# Preservation of the Teeth

**Published:** 1849-07

**Authors:** 


### PRESERVATION OF THE TEETH.

EXTRACT FROM THESIS OF R. T. WILLARD, GRADUATE OF O. C. DENTAL SURGERY, SESSION 1848-9.

Perfection characterizes all the works of the Great Architect of the Universe. It matters not whether we trace its exhibition in the flying worlds that traverse boundless space, the harmonious concert in which they move, and the great law of attraction which governs all their motions, or whether we view its manifestations in some of the myriads of nature's minutest structures, still we find its presence. No where is its exemplification more beautiful than in the structure of man, the noblest of God's works. In him we discover the most perfect adaptation of all the parts, to the performance of the particular functions for which they were designed—each part depending upon the other's for assistance in the accomplishment of its own appropriate end, thus forming an inseparable, and perfect whole, of which no organ, or set of organs, can be deranged, without producing injurious effects upon others, and through them, upon the whole system; hence we see the necessity of preserving in a perfect condition all parts of the body, and among these the teeth will at present claim our attention.

It is a singular fact, that the teeth, though the most dense and hard portion of the body, are the most liable to decay; and they are almost the only structure that does not possess recuperative powers, so that when they are destroyed by disease or otherwise, their loss is total, and no subsequent operation of the economy will replace them.

Notwithstanding the high degree of perfection which the science of Dentistry has attained, yet no artificial substitute which can be made is equal to the perfect organ, as supplied by nature, for the purpose of mastication, and the various uses for which the teeth are designed. So apparent is this, that it would seem unnecessary to speak on this subject, supposing that the difference in this particular, between nature and art, and the superiority of the former, would be appreciated and understood by all; but the fact that thousands are passing along, regardless of the condition of the teeth, letting them go to destruction without even the slightest effort for their preservation, would seem to prove that they either do not appreciate their value, nor anticipate their loss, or else they do not understand the means of preserving them.

In pointing out the means of preserving the teeth in a healthy condition, it may be necessary to briefly notice some of the diseases to which they are most liable, and the manner in which they operate to produce such injurious effects as they sometimes do. Unless the disease, and the causes which operate to produce it are understood, there can be but little prospect of success in an attempt to prevent or remove the disease; but if we correctly understand the causes and their *modus operandi*, in the production of disease, and where these causes are under our control as they generally are, in diseases of the teeth, we can go to work upon philosophic principles, and with almost mathematical certainty, for the prevention or removal of disease.

In briefly alluding to a few of the diseases of the teeth, *Caries* will first come under consideration. The terms gangrene and mortification, have been used to express the same condition of disease, but caries is now generally used, and the term is as good as any other. It is a disease that may attack all teeth, and any portion that is exposed, or not covered by the gums.

As to the causes of caries it may be remarked, that it is pretty generally admitted that they are external, and principally chemical. Notwithstanding the many proofs that may be adduced in favor of this theory, yet there are some who still doubt its correctness, and urge among their objections, that the teeth of the upper jaw are more liable to decay than those of the under, the reverse of which they say should be the case, was the cause chemical, for the saliva and any other chemical agents that may be in the mouth, would naturally collect in the under jaw, and thus come in contact with the lower teeth, and act more readily upon them than upon the upper ones. They must, however, recollect that in order to produce any decided injurious effect upon the teeth, a substance must be kept for some time in contact with them, and in the lower jaw, all foreign matter is much more readily washed away from the teeth by the tongue and saliva, than in the upper jaw. Again, they say they cannot understand how the theory of external causes will explain the fact, that the teeth more frequently decay on their labial than on their lingual surface. Now it is plain that the same explanation is applicable here as before; the motions of the tongue upon the inside of the mouth, will keep particles of foreign matter from remaining in contact with that side of the teeth, and thus their injurious action is prevented; and upon no other principle can they equally well explain why caries occur so much more frequently upon the approximal edges and outer surface, than upon the internal surface of the teeth. It is evident that particles of food and other substances introduced into the mouth, would be more readily retained between the teeth, and on their outside than internally, hence their action is more injurious and apparent.

There is one other circumstance which has been urged against this theory. It is, that the teeth of the female decay more frequently than those of the male—whereas they say they are both exposed to about the same influences. According to Simon, chemical analysis has shown that the teeth of the female contain a greater proportion, by from three to six per cent. of the carbonate of lime, and correspondingly less of the phosphate than those of the male.

Now there are few acids, perhaps but a single one, the hydrocyanic, that will not act readily upon the carbonate, whereas there are comparatively few that will act readily upon the phosphate. So this difference may be satisfactorily accounted for, on the ground that the teeth of the female contain a greater proportion of the carbonate of lime, which more readily unites with the acids of the mouth, and thus causes the teeth to decay.

Again, others object to the theory of external causes, and with some degree of plausibility, urge the analogy between the caries of the teeth and caries of other bones, the operating causes of which must generally be internal, and the vascularity and vitality of the teeth following the same laws as in other bones, render them liable to inflammation, which like other parts may result in caries, or the complete death of the part.

Now that internal causes *never* produce caries, I shall not attempt to prove, nor, like many of our best authors of the present day, entirely deny—but the fact that the observations of a large majority of practitioners of modern times, whose opinions are worthy of the highest credit and respect, have so universally gone to prove, that the causes operating to produce caries of the teeth, are external, that it is very generally admitted. When we consider the complete and almost perfect control which we possess over this disease, and also the kind of treatment by which its progress is arrested, and its commencement prevented, we can scarcely avoid the conclusion that the causes we are combating are external.

Assuming, then, that this theory is correct in most, if not all instances of decay of the teeth, we are prepared to understand how the present mode of treatment is attended with beneficial results—but if the causes were altogether internal, we might anticipate injurious rather than salutary effects to follow from such treatment, and in this case very limited indeed would be the usefulness of the dentist. If this view be correct, and the external causes are, foreign matter, as acid in contact with the teeth, the indication for their preservation is at once clear, and consists in *keeping them clean.* Teeth that are perfectly developed, regular, and well arranged, if kept perfectly clean, and free from all foreign and unnatural matter in the mouth, will seldom, if ever decay, or require the assistance of the Dentist. Circumstances, and the arrangement of the teeth, however, sometimes render it almost if not quite impossible to keep them in a proper condition, yet this should not prevent every effort being made that will in the least contribute to their preservation.

As "prevention is always better than cure," it is a matter of great importance that preventative means be always first used, that the fell destroyer, disease, may not even obtain a foothold upon which to dispute the ground with the rightful possessor—health.

To keep the teeth clean is a much easier matter than many suppose, although it requires the strictest attention, the neglect of which often renders it an exceedingly difficult operation, perhaps requiring the assistance of the Dentist.

If, however, the teeth by neglect have already become decayed, and caries already commenced its ravages, which if not arrested will soon result in the total destruction of these invaluable organs, it is necessary that immediate and thorough means be employed to preserve them from further injury by the action of the disease. For the accomplishment of this, science proposes but a single mode of treatment, which consists in the entire removal of all the diseased portion of the tooth, and when a cavity remains, perfectly filling it with some substance that will entirely prevent the action of foreign substances upon the inner structure of the tooth. For this purpose gold is decidedly superior to every other substance known. When thus thoroughly treated, a tooth that had commenced decaying, may be preserved for years, or even during life, in a good and serviceable condition, which would otherwise soon have been entirely destroyed.

